# Prevention services via public long-term care insurance can be effective among a specific group of older adults in Japan

**DOI:** 10.1186/s12913-021-06495-0

**Published:** 2021-05-30

**Authors:** Tomoko Ito, Takahiro Mori, Hideto Takahashi, Natsumi Shimafuji, Katsuya Iijima, Satoru Yoshie, Nanako Tamiya

**Affiliations:** 1 Department of Health Services Research, Faculty of Medicine, University of Tsukuba, 1-1-1 Tenno-dai, Tsukuba, Ibaraki, 305-8575 Japan; 2 Health Services Research and Development Center, University of Tsukuba, 1-1-1 Tenno-dai, Tsukuba, Ibaraki, 305-8575 Japan; 3 Department of General Medical Science, Graduate School of Medicine, Chiba University, Chiba, Chiba Japan; 4 Department of General Internal Medicine, Eastern Chiba Medical Center, Togane, Chiba Japan; 5 National Institute of Public Health, 2-3-6 Minami, Wako, Saitama, 351-0197 Japan; 6 Ridgelinez Limited, 2-6-1, Marunouchi, Chiyoda-ku, Tokyo, 100-6922 Japan; 7 Institute of Gerontology, The University of Tokyo, 7-3-1 Hongo, Bunkyo-ku, Tokyo, 113-0033 Japan; 8 Institute for Future Initiatives, The University of Tokyo, 7-3-1 Hongo, Bunkyo-ku, Tokyo, 113-0033 Japan; 9 Department of Health Policy and Management, School of Medicine, Keio University, 35 Shinano-cho, Shinjuku-ku, Tokyo, 160-8582 Japan

**Keywords:** Prevention services, Community care, Older adults, Long-term care insurance

## Abstract

**Background:**

To evaluate the effects of prevention services provided by long-term care insurance (LTCI) for older adults who require support from LTCI in Kashiwa City, Japan.

**Methods:**

We conducted an analysis using the following population-based longitudinal data in Kashiwa City between April 2012 and March 2015: Data of National Health Insurance and LTCI claims, the survey for certification of LTCI, the register, and premium tier classification. All data was linked using the pre-assigned anonymous identifying numbers. We analyzed the Cox regression model using the time for the deteriorations of levels of certified care need in LTCI as an outcome and the use of preventive care services as the primary exposure among participants aged 75 years or older, who had either support levels 1 or 2 at the beginning of this analysis. The study was further stratified by both age and initial support level.

**Results:**

The final analysis included 1289 participants. The primary result showed, among all participants, that preventive service was not effective (hazard ratio 0.96, 95% confidence interval 0.78–1.19). In our sub-analysis, the preventive service was effective in avoiding deteriorations only among those aged 85 and older with support level 1 (HR 0.65, 95% CI 0.43–0.97) out of four groups.

**Conclusions:**

The preventive services of LTCI in Kashiwa City showed a significant effect on the deterioration among subjects aged 85 or older, whose disability level were low (support level 1). Our results suggest that the prevention services provided by LTCI may not be effective for all older individuals; to provide these services efficiently, local governments, as insurers of LTCI, will need to identify the specified groups that may benefit from the preventive services. Additionally, it is necessary to re-examine what preventive interventions may be effective, or redesign the health system if necessary, for those who were not affected by the intervention.

## Background

Many societies are rapidly aging. Japan began to see a super-aging population earlier than other countries and is expected to see a peak in their aged population in 2025 [1], when one third of individuals in Japan will be aged over 65. In 2000, long-term care insurance (LTCI) was introduced in Japan to be used as a public insurance system to provide care for disabled older adults, both at home and in facilities [2, 3]. As Japanese society has had an increasing number of aging adults, the number of disabled people in LTCI has also increased greatly. In 2006, the number of individuals on LTCI had increased to 2.17 million, which is approximately 200% more than when the initiative was announced [4]. In particular, increases in the numbers of people who were certified as having mild disabilities, meaning they needed low levels of care was noticeable. Many of those who needed a low level of care were considered to have “disuse syndrome (inactive lifestyle),” where daily function gradually declines due to conditions such as falls, bone fractures, and joint diseases. In 2006, the government established prevention services designed to decrease the number of people becoming disabled [5]. Prevention services include day care (rehabilitation), day services (support for daily living), and visiting services. These services are arranged for each person based on an assessment of their disabilities. In the regular reforms, provision of prevention services was gradually left in the hands of local municipalities [6] who may change or limit the offered prevention services. Therefore, discussion is necessary to plan and implement future prevention services and determine how resources will be concentrated.

The effects of prevention services have been evaluated in several studies. Many showed the effects of specific programs such as muscle strength rehabilitation and locomotive function improvement programs [7–11]. To evaluate the outcomes, these studies used specific physical functions such as the Timed Up Go test [7–9, 11, 12] or time standing on one leg [7–10]. When discussing public health policies, indicators are expected to address items such as supporting the level of certified care need in LTCI. However, few studies used that level as an outcome [12–16] and their results were controversial [13–16].

Ensuring that public health insurance is sustainable is an extremely difficult task for countries [17]. In general, the allocation of resources should be more efficient while discerning the policy effects. At present, there are not sufficient research results that would allow policymakers to make decisions about the preventive services offered in LTCI. The research informing these decisions should be based on public data that can be accessed by any local municipality. In addition, the outcome indicator should be an official one that is widely used, such as the certification level in LTCI. Therefore, we aimed to evaluate the effect of prevention services on disabled adults using the level of certified care need in LTCI. To offer further insight into resource investment, we showed the difference in the preventive effects on age and the initial certified level. This study’s results could demonstrate which populations are more efficient targets for LTCI preventive services. This method may be usable in all municipalities in Japan by using the same data.

## Methods

### Study design and data collection

We conducted a population-based retrospective longitudinal analysis in Kashiwa City, Prefecture of Chiba, Japan. In this study, we used data of the National Health Insurance (NHI), LTCI claims, survey for certification of LTCI, registry, and insurance fee level for Kashiwa City between April 2012 and March 2015. Kashiwa City is located in the Kanto region and has a population of 400,000 and its proportion of people aged 65 and older is 24.4%, which is lower than the national average of 26.6% in 2015. The LTCI system in Japan insures adults aged 65 and over, as well as those who are over 40 years old and have specific diseases [2]. When insured individuals need long-term care, they can use a survey to apply to have the service certified as required care; this is considered the necessary qualification to use the services. The certification survey is conducted by a surveyor who is commissioned by the local government to visit the subject and observe them directly. This survey was conducted almost nationwide with some differences, and has been statistically processed. All data was linked using the ID number assigned for the study. This individual linked dataset was used in some previous studies [18–21].

The data was provided by Kashiwa City to the University of Tsukuba for research purposes, and the data provided and used for research purposes was approved by the Medical Ethical Review Board of University of Tsukuba (approved number 1448).

### Participants

Participants were individuals who were 75 years old or older living in Kashiwa City and who first received the LTCI certification from July 2012 to March 2014. Those who were certified as support level 1 or 2 were included in the study. Their status as residents of Kashiwa City was confirmed from the Basic Resident Register data before the first certification that they required long-term care. In addition, in order to demonstrate that the use of preventive care services for 6 months after the initial certification was necessary, it was also confirmed that participants were both alive and had remained at support level 1 or 2 during the 6 months after their certification. Those who had been hospitalized during the 6 months were excluded because their hospitalization limited their use of LTCI services during the time period.

### The certification levels for LTCI services in Japan

The degree to which individuals need LTCI services indicates their needs for care in daily life; this relates to the amount of LTCI services and available services they will use [22]. In 2006, the certified levels for LTCI were revised and divided into seven categories: support levels 1–2 and care levels 1–5, with care level 5 requiring the most intense amount of care [5]. These levels are determined by the survey and defined by the estimated amount of care time [23]. The LTCI survey collected 74 basic items and as well as special notes about caregiving situation. The items included their ability for daily living tasks like “getting up and walking”, or “bathing, toileting, and eating.” The family should also be present at the survey so that the individuals’ daily lives can be investigated in detail.

The clinical picture of an individual requiring care level 5 is almost bedridden. Those with care levels of 4 or 5 received services for toileting, eating, and bathing, while those with care levels 1 to 3 often received services for house cleaning, laundry, or watching to ensure that they did not wander. Specifically, individuals with support level 1 can perform most of the basic activities of daily living on their own but need to remain active to prevent the deterioration of their current state, increasing their need for nursing care. This category does require some support, though. Those who were certified as support level 2 require more assistance to complete activities of daily living, and some nursing care is also required. Based on the policy objectives, it is expected that their condition will remain the same or improve using LTCI services [6].

Support levels 1 and 2 were introduced in the 2006 reform of the LTCI system to enhance preventive interventions. In this reform, individuals certified as needing more than 25 min, but less than 32 min of care remained at support level 1. However, those who had been care level 1 who needed 32 min but less than 50 min of care, and whose condition was expected to improve with care, were changed to support level 2. This change was based on examining the possibility of maintaining or improving the condition [23]. Therefore, people with support level 2 then to have a smaller need for care and have the possibility of improving through preventive interventions. Finally, the distinction between support level 1 and 2 is based on the minutes of care they need.

### Outcome

The outcome variable was the time (unit: days) from the date on which the participant was first certified for LTCI use, for either support level 1 or 2, until their state deteriorated to care level 1 or greater. Care level 1 indicates the elderly who need more services than watching-over care, especially those with limited mobility. For those who did not show any deterioration, the end date of the validity period in their observed final certification of support level 1 or 2 was considered as the observation end date and censored. To consider a selection bias, we checked that observation of participants was complete. The Basic Resident Register data was also used to confirm that no participants had died or moved before the end of the observation period.

### Exposure

Exposure was based on the use of preventive services for 3 months, including the first month when participants were certified as eligible LTCI user, which was designated as support level 1 or 2. Those who used prevention services at least once were included in the exposure group. Long-term care prevention service is an LTCI service that can be used by those who have been identified as support level 1 or 2 and aims to ensure that the participant’s independence in daily life does not deteriorate. The content of visiting care includes bathing, nursing, or outpatient care and rehabilitation. Home visit nursing for prevention was used mostly to gain advice and treatment for their conditions or rehabilitation [24]. Also, they had activities with other users and muscle training through outpatient services. Coordination of preventive care services is performed by specialized staff commissioned by the local government [5]. However, the use of services is voluntary and entirely dependently on the agreement of the individuals or their families. It is predicted that the use of the service is influenced by individual characteristics including economic status. Therefore, it is necessary to adjust with covariates.

### Covariates

Covariates were used to indicate subject severity. Covariates were set based on the use of medical services in the 3 months before the first certification month. The Charlson Comorbidity Index (CCI) scores calculated from the illness information and included in the medical claim data were treated as a covariate [25]. We used the revised CCI scores as validated for Japanese administrative data [26]. In addition, the degree of independence in daily activities and in cognitive function shown in the survey data for LTCI certification were treated as covariates, and were found to be predictors of deterioration in the previous analysis [27]. The degree of independence in daily activities or in cognitive function is widely used in Japan to evaluate elderly activity (Appendix 1). These two indexes are created while the investigator objectively determines the degree of independence regarding the state of the elderly with disabilities at the care site. In the index for elderly daily activities evaluation, the following four levels of judgment criteria are used: Rank J (living independently), Rank A (pre-bedridden, but able to get out of bed for the day), Rank B (bedridden, but able to sit up), and Rank C (bedridden). For our model, we defined the disabled status was dependent when the degree of independence in daily activities was Rank A or severer. For those who certified as support 1 or 2, it is presumed that they do not need a lot of care. However, in Japan, the level of certification is based on the time required for care. In the present study, a slight difference in activities of daily living could be expected within the study population. The present index for elderly daily activities evaluation includes only one category for living independently. As we were limited by the use of this index, the divisions of independent (Rank J) and others (Rank A or more severe) were considered to detect such differences. On the other hand, the degree of independence in cognitive function includes nine stages of daily life independence of elderly people with dementia. The dependence for demented status in our model was defined dependent when that cognitive index was Rank II or more dependent. For covariates in the socio-economic situation, we used the insurance fee of LTCI. The fee level is calculated based on the taxation of the insured households, with tax-exempt households designated as low-income households. The previous study showed that income level influences service use [20].

### Statistical analysis

We showed the characteristics of participants among preventive services users and non-users with statistical tests. Chi-square tests were performed on the binary variables and Wilcoxon’s sum rank test were applied to the ordered variable (the CCI). Also, we drew the survival curves showing the deterioration from support level 1 or 2 to care level 1 or worse with Kaplan-Meier estimates. The differences of the curves were tested with log-rank tests. We analyzed the Cox regression model using the time from the first day of certification for LTCI to when the participant’s condition had deteriorated to certified care level 1 or worse as an outcome variable. The exposure was the use of preventive care services, adjusted for covariates. Primary analysis was conducted in all subjects. These models were also tested in four groups categorized according to age (< 85, > = 85) and the first support level (support level 1, 2) as sub-analysis. The proportional Hazard assumption in Cox Model was examined using the Schoenfeld Residuals Test. STATA Version 14.2 (Stata-Corp LLC, College Station, Texas, United States of America) was used for the analysis. The statistical significance level was a two-sided *p*-value of less than 5%.

## Results

**Fig. 1:**
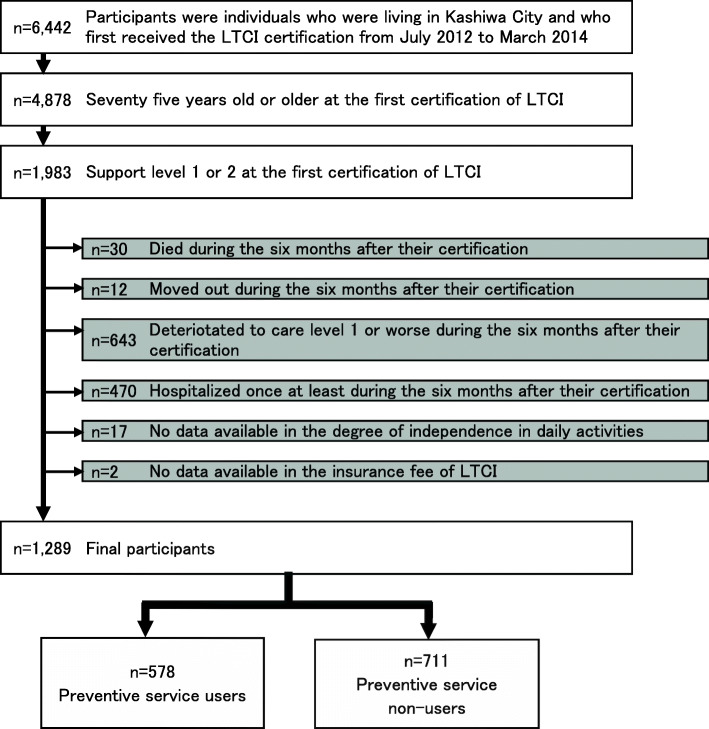
Participants flow

The study subjects included 1289 participants (Fig. 1), of which 578 participants (44.8%) were preventive service users. The participant characteristics are shown in Table 1. Only sex, the initial certified level, and income level were significant related to groups. Of those studied, 39.9% of men and 47.0% of women used prevention services (*p* = 0.018); 41.9% were initially certified as support level 1 and 50.3% at of support level 2 (*p* = 0.004); finally, 47.5% of those with low income and 39.4% of those with high and middle income used services (*p* = 0.006). During the observation period, 348 (27.0%) of all participants showed a deterioration in their certificated level to care level 1 or worse. Among service users, the median time to deterioration was 212 days, with a minimum of 0 days, a maximum of 789 days and an interquartile range of 189 days. In the non-users, the median time to deterioration was 212 days, with a minimum of 8 days, a maximum of 735 days and an interquartile range of 133 days. The proportional Hazard assumption in Cox Model was satisfied.

**Table 1:** Differences in covariates between preventive service users and non-users

		Preventive service users		Non-users		Total	
		(*n* = 578)		(*n* = 711)		(*n* = 1289)	
		n	%	n	%	n	p-value
Age	< 85	331	44.2	418	55.8	749	0.581
	> = 85	247	45.7	293	54.3	540	
Sex	Male	159	39.9	239	60.1	398	0.018
	Female	419	47.0	472	53.0	891	
Certified level	Support level 1	354	41.9	490	58.1	844	0.004
	Support level 2	224	50.3	221	49.7	445	
Use of inpatient medical service	Yes	397	44.3	499	55.7	896	0.561
	No	181	46.1	212	53.9	393	
Use of outpatient medical service	Yes	411	44.8	507	55.2	918	0.937
	No	167	45.0	204	55.0	371	
Disabled status	Dependent	221	46.4	255	53.6	476	0.381
	Independent	357	43.9	456	56.1	813	
Demented status	Dependent	108	44.8	133	55.2	241	0.992
	Independent	470	44.8	578	55.2	1048	
Household income	Low	410	47.5	453	52.5	863	0.006
	High and Middle	168	39.4	258	60.6	426	
Charlson Comorbidity Index scores	=0	184	43.7	237	56.3	421	0.843*
	> = 1	232	45.6	277	54.4	509	
	> = 3	123	48.2	132	51.8	255	
	> = 5	39	37.5	65	62.5	104	

* *p*-value by Wilcoxon’s sum rank test

**Fig. 2:**
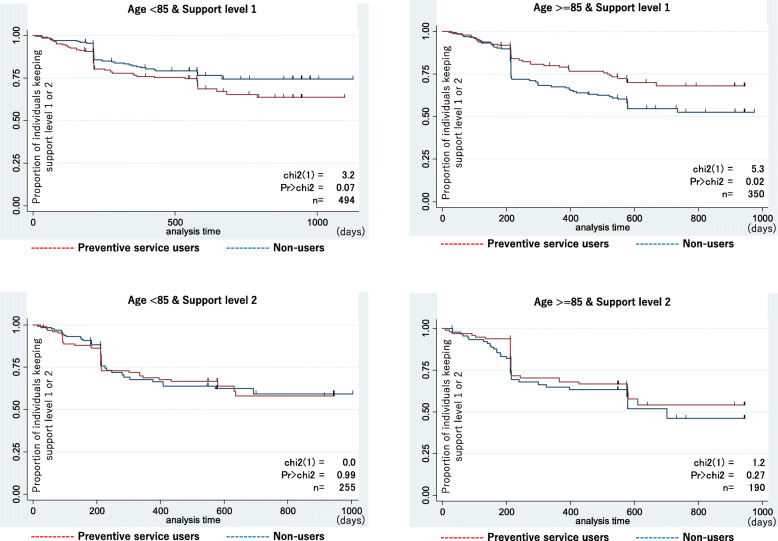
Survival curves of deterioration to care level or worse in preventive services users and non-users in sub-groups by age (< 85 y.o., ≥85 y.o.) and support level (1, 2)

According to the results of primary analysis using the Cox regression model and including all covariates, the preventive service was not effective (hazard ratio 0.96, 95% confidence interval 0.78–1.19) (Table 2). In that model, only older age (age > =85), more severely disabled (support level 2), and dependency for demented status were significant factors of deterioration in the primary analysis. More severe dementia status was the most influencing factor for deterioration. However, Table 3 shows the sub-analyses in which we performed Cox regression stratified by age and initial care need status where the preventive service showed effectiveness (hazard ratio 0.65, 95% confidence interval 0.43–0.97) in only one group (age > =85 & support level 1) of the four groups. In Fig. 2, there was a significant difference between survival curves of preventive service users and non-users only in the group (age > =85 & support level 1). Among two groups of support level 2, the curves were duplicated and there were no significances. On the other hand, in the group (age < 85 & support level 1), the deterioration among preventive service users were more than non-users, though not significant.

**Table 2:** Effects of preventive service to avoid deterioration to care level 1–5 from support level 1–2 as certification of LTCI system in Japan

		Adjusted				
		HR	95%CI			Proportionality^a^
Preventive service	Use	0.96	0.77	–	1.19	*P* > 0.05
	No use	1.00				
Age	< 85	1.00				
	> = 85	1.26	1.01	–	1.56	P > 0.05
Sex	Male	1.00				
	Female	0.76	0.57	–	1.03	P > 0.05
Certified level	Support level 1	1.00				
	Support level 2	1.47	1.17	–	1.84	P > 0.05
Use of inpatient medical service	Yes	1.64	0.56	–	4.85	P > 0.05
	No	1.00				
Use of outpatient medical service	Yes	0.68	0.23	–	2.01	P > 0.05
	No	1.00				
Disabled status	Dependent	1.12	0.90	–	1.40	P > 0.05
	Independent	1.00				
Demented status	Dependent	2.46	1.95	–	3.11	P > 0.05
	Independent	1.00				
Household income	Low	0.88	0.65	–	1.17	P > 0.05
	High and Middle	1.00				
Charlson Comorbidity Index scores	=0	1.00				
	> = 1	0.85	0.65	–	1.13	P > 0.05
	> = 3	0.99	0.72	–	1.36	P > 0.05
	> = 5	0.92	0.61	–	1.38	P > 0.05

*HR* hazard ratio, *95% CI* 95% confidence interval

^a^ Schoenfeld Residuals Test

**Table 3:** Effects of preventive service to avoid deterioration to care level 1–5 from support level 1–2 as certification of LTCI system in Japan

		No adjusted				Adjusted^a^				
		HR	95%CI			HR	95%CI			Proportionality^b^
Age < 85 & Support level 1	(*n* = 494)	1.43	0.96	–	2.13	1.33	0.89	–	1.99	P > 0.05
Age < 85 & Support level 2	(*n* = 255)	1.00	0.64	–	1.58	1.16	0.71	–	1.88	P > 0.05
Age > =85 & Support level 1	(*n* = 350)	0.63	0.43	–	0.94	0.65	0.43	–	0.97	P > 0.05
Age > =85 & Support level 2	(*n* = 190)	0.76	0.47	–	1.24	0.84	0.51	–	1.39	P > 0.05

^a^Adjusted for sex, use of inpatient and outpatient medical service, disabled status, demented status, income, and Charlson Comorbidity Index scores

*HR* hazard ratio, *95% CI* 95% confidence interval

^b^ Schoenfeld Residuals Test

## Discussion

In the present study, we analyzed the effects of prevention services to prevent or impede the deterioration in the level of certified care need of LTCI in Japan. The prevention services were effective only in those aged over 85 and needing mild care. In the other groups, use of the prevention service did not maintain the level of certified care needed. Previous studies examined the effects on only specific aged groups [15], and did not conduct analysis with varying aged groups. Our results showed the effects of preventive services in comparison with varied age and status of disability. Globally, the prevention strategy for the risk of falls is beginning to be questioned for its effectiveness [28]. Cochrane Library or other literature reviews could show only limited effectiveness with slightly difference from control group [29, 30]. Our study could show this possibility of an additional limited effect on these trends. This would be good starting point for considering more efficient service placement that is more tailored to the characteristics of the target population.

Our findings indicate that prevention services may not be effective for all subjects who were first certified as support levels 1–2 between the ages of 75 and 85, and for those who were certified as support level 2 at age 85 or older. Prevention service of LTCI in Japan was initially started as a “preventive care benefit” based on uniform national standards and rewards. From April 2015 onward, the two preventive services (visiting and outpatient care) were transferred to the “Community support program” operated by local governments. For these two services, the financial structure is the same as that for preventive benefit. However, each local government can decide its own standards and rewards. Services can be operated in various forms such as services with more flexible standards and services by residents, including volunteers. For local governments, it is necessary to consider more effective resource allocation in the future when available resources become limited. Our results can allow governments to consider which groups or characteristic segments should be targeted for effective intervention. We demonstrated the potential effects of preventive services for some groups of people. The local government as an insurer of LTCI must identify the groups that may benefit from preventive services in their areas. In addition, it is necessary to re-examine what is an effective preventive intervention for those who did not show efficacy. This is also a need to redesign the system for municipalities where much of the preventive work has been delegated. They have the duties of both efficient resource allocation and the health of older adults. In order to do so, future studies that can provide a clear mechanism for why the intervention was or was not effective are needed. Furthermore, exploratory fieldwork would be necessary to find the real needs among citizens. That fieldwork could enhance the policymaking to change from a top-down to a bottom-up system, as suggested by a previous study [31].

Older age was a factor of deterioration in previous studies [15, 32]. In some studies with multivariable analysis, older age was a significant factor of deterioration in the adjusted model [15], which is consistent with our results in the primary analysis (Table 2). No previous study has analyzed the effects of avoiding their deterioration in the very older-aged group using the public claim data. In our results, preventive services were effective only in older ages and for mildly disabled individuals. This significant effect of prevention services seemed to depend on the potential characteristics among those who needed care services for the first time. The subjects aged 85 and over without certification were expected to spend their lives with small disabilities or health problems. In that group (age > =85 and support level 1), there are many persons who are potentially healthier than those in other groups [33] Therefore, it seems that this group was likely to have an effect of preventive care.

Although the age of 85 years is common as a delimiter for the 5-year age categories, we further investigated the threshold of the cutoff age by changing the age from 81 to 89 by 1 year. Specifically, we analyzed the Cox regression model with fully adjusted in the groups defined by the reclassified age variable. We confirmed the effect of prevention services was not significant when the cut-off age was 81–84 years old. We also found there was a significant effect when the cut-off age was 85–87 years old, but not when the cut-off age was 88 or 89 years old. However, due to the possibility of the lack of power by decreased sample sizes, it is difficult to speculate any further whether the prevention service is more effective or not for those who are 88 years old or over. Studies with lager sample size would be warranted to address such question.

The strength of our study is that it uses public claim data. Using public administrative data allowed us to follow the entire population of one city, unlike some previous studies that adopted a pre-post intervention of specific groups as their study design [8, 9, 13]. In this study, we collected information on the use of medical services and co-morbidities for adjustment using NHI claim data. Further, we extracted the status of cognitive impairment from the certification survey data, which included the factors of deterioration in the previous analysis [27]. This variable collection across data became possible only by linking several administrative data between individuals. Our study was the first to use linked administrative data for evaluating preventive services.

### Limitation

There were several limitations to be considered in the present study. First, we defined the preventive services provided only during the 6 months after participants’ initial certification of LTCI. The effect of preventive service provided at any time was not examined correctly. Therefore, there was some limitation in the time of service provision. We focused on the first duration after first certification because we expected that during this period, their needs would be new, thus motivating them for service use. Even for local government experts, such a duration of service use enables accurately considering subjects’ needs.

Second, this study focuses on only one location. Therefore, the effects of the characteristics and resource allocation of the participants based on the location must be considered. However, this study is the first to evaluate preventive services based on the certified level of LTCI using insurance claim data. Our study reveals useful methods and findings for future local government-based interventions.

Third, the first certification of LTCI was defined based on the local government records during the observed period. The individual identification depended on the insured person number that was applied by the local government. The insured person number was changed when the person moved from one location to another. Therefore, it was impossible to identify whether the person had LTCI certification before moving. This limitation also affects the use of the claim data, even if it was nationwide. Such restrictions on individual tracking are difficult unless individual numbers are assigned to each citizen, and the countermeasures will be an issue for future studies.

Fourth, those who used prevention services at least once were included in the exposure group regardless of the frequency, types, or combinations of services, making it difficult to determine whether and how these components contributed to the observed effects. There is some speculation on the definition of exposure in present study. This exposure may vary greatly from individual to individual. In this study, we only defined exposure as comprehensive service use using claim data. Therefore, other definitions of exposure, such as frequency and details of services used, may change the results and should be considered. Future research should be conducted to examine the effects of such service usage in details.

Fifth, there might be a self-selection bias because the use of LTCI services depends on the free decision of the users and their family. Therefore, we should consider the bias between this study’s subjects and the overall targeted population for the hypothesis.

Finally, the effects of unknown confounding cannot be denied. We tried to gather covariates using official data from the local government; nonetheless, there remains the possibility of handling other covariates by linking more existing data such as socioeconomic status, family status, or educational history. This necessitates future research involving analysis of more covariates for precise results.

## Conclusion

n conclusion, the preventive services of LTCI in Japan showed a significant effect on deterioration only among subjects aged 85 or older and whose overall support needs were low. These individuals were expected to have a great possibility of prevention deterioration than other very older adults. The service provision focusing on these individuals can be efficient. To achieve an efficient allocation of limited LTCI resources, it is important for policymakers to identify the population to be targeted for prevention services. Meanwhile, it is necessary to re-examine interventions that may be preventive for those who did not show efficacy or to redesign the system to support the health for those who were not affected. Also, this study can encourage analysis based on public data that can be handled by any local municipalities for their policymaking decisions.

## Data Availability

No additional data are available due to data protection requirements.

## Appendix

Appendix 1 The degree of independence in daily activities or in cognitive function.

**Table:**

**The degree of independence in daily activities**	
Rank J	(living independently) A person who has a physical disability (due to sickness aftereffects) but is almost independent of daily life and can go out alone.
	1. Go out using transportation
	2. Go out to the neighborhood
Rank A	(pre-bedridden) People who can do their daily routine indoors by themselves, such as eating, toilets, and changing clothes, and need help from a caregiver when going out to the neighborhood.
	1. Go out with assistance and live mostly out of bed during the day
	2. You rarely go out and sleep or wake up during the day
Rank B	(bedridden) People who spend most of their day in bed and need help from a caregiver in any of their meals, toilets, or changing clothes.
	1. Transfer to a wheelchair and eat and excrete away from bed
	2. Transfer to a wheelchair with assistance
Rank C	(bedridden) A person with a higher degree of disability than Rank B and who needs help from a caregiver for eating, toilets, and changing clothes.
	1. Roll over by yourself
	2. I can’t turn over by myself
**The degree of independence in cognitive function**	
Rank I	People who have cognitive symptoms but who are almost independent of their daily lives both at home and socially.
Rank II	A person who has some behaviors and communication difficulties that interfere with daily life, but who can stand on their own if someone is watching over them.
Rank IIa	Those who have the above condition II outdoors. Lost way, mistakes in shopping and money management, etc.
Rank IIb	Someone in the house who has the above condition II. Drugs cannot be managed, visitors cannot respond, etc.
Rank III	People who require nursing care due to symptoms such as behavior that interferes with daily life or difficult communication.
Rank IIIa	Those who are mainly in the above state during the daytime. Can’t eat/toilet/change clothes well, put things in mouth, wander, incontinence, strange voice, etc.
Rank IIIb	People who are in state III above at night. Symptoms are the same as IIIa.
Rank IV	People who have frequent behaviors that interfere with daily life, communication is difficult, and need constant care. Symptoms are the same as III.
Rank M	Persons with significant mental symptoms or severe physical illness who require specialized medical care. Delirium and excitement, problematic behaviors resulting from them, etc.

## Abbreviations

LTCI: Long-Term Care Insurance
HR: Hazard Ratio
CI: Confidence Interval
NHI: National Health Insurance
ID: identification
CCI: Charlson Comorbidity Index
LLC: Limited Liability Company
